# Maize microarray annotation database

**DOI:** 10.1186/1746-4811-7-31

**Published:** 2011-10-01

**Authors:** Nanette Coetzer, Alexander A Myburg, Dave K Berger

**Affiliations:** 1 Bioinformatics and Computational Biology Unit, Department of Biochemistry, University of Pretoria, Private Bag X20, 0028, South Africa; 2 Department of Genetics, Forestry and Agricultural Biotechnology Institute (FABI), University of Pretoria, Private Bag X20, 0028, South Africa; 3 Department of Plant Science, Forestry and Agricultural Biotechnology Institute (FABI), University of Pretoria, Private Bag X20, 0028, South Africa

**Keywords:** Agilent, maize, microarray, annotation, B73

## Abstract

**Background:**

Microarray technology has matured over the past fifteen years into a cost-effective solution with established data analysis protocols for global gene expression profiling. The Agilent-016047 maize 44 K microarray was custom-designed from EST sequences, but only reporter sequences with EST accession numbers are publicly available. The following information is lacking: (a) reporter - gene model match, (b) number of reporters per gene model, (c) potential for cross hybridization, (d) sense/antisense orientation of reporters, (e) position of reporter on B73 genome sequence (for eQTL studies), and (f) functional annotations of genes represented by reporters. To address this, we developed a strategy to annotate the Agilent-016047 maize microarray, and built a publicly accessible annotation database.

**Description:**

Genomic annotation of the 42,034 reporters on the Agilent-016047 maize microarray was based on BLASTN results of the 60-mer reporter sequences and their corresponding ESTs against the maize B73 RefGen v2 "Working Gene Set" (WGS) predicted transcripts and the genome sequence. The agreement between the EST, WGS transcript and gDNA BLASTN results were used to assign the reporters into six genomic annotation groups. These annotation groups were: (i) "annotation by sense gene model" (23,668 reporters), (ii) "annotation by antisense gene model" (4,330); (iii) "annotation by gDNA" without a WGS transcript hit (1,549); (iv) "annotation by EST", in which case the EST from which the reporter was designed, but not the reporter itself, has a WGS transcript hit (3,390); (v) "ambiguous annotation" (2,608); and (vi) "inconclusive annotation" (6,489). Functional annotations of reporters were obtained by BLASTX and Blast2GO analysis of corresponding WGS transcripts against GenBank.

The annotations are available in the Maize Microarray Annotation Database http://MaizeArrayAnnot.bi.up.ac.za/, as well as through a GBrowse annotation file that can be uploaded to the MaizeGDB genome browser as a custom track.

The database was used to re-annotate lists of differentially expressed genes reported in case studies of published work using the Agilent-016047 maize microarray. Up to 85% of reporters in each list could be annotated with confidence by a single gene model, however up to 10% of reporters had ambiguous annotations. Overall, more than 57% of reporters gave a measurable signal in tissues as diverse as anthers and leaves.

**Conclusions:**

The Maize Microarray Annotation Database will assist users of the Agilent-016047 maize microarray in (i) refining gene lists for global expression analysis, and (ii) confirming the annotation of candidate genes before functional studies.

## Background

Currently, there are several maize microarray platforms available, including an Affymetrix short oligonucleotide array [1], a Nimblegen 50-mer array [2], a 70-mer array from the University of Arizona Maize Oligonucleotide Array project [3] and the 60-mer Agilent-016047 Maize 4 × 44 K microarray [4].

The Agilent microarray platform [5] is a mature technology that yields high quality gene expression data, which can be readily analyzed using established statistical tools [6]. The Agilent-016047 Maize 4 × 44 K microarray was custom-designed by the Walbot laboratory, with 42,034 *in situ *synthesized 60-mer oligonucleotide reporters (excluding controls) [7]. Currently, the Agilent "e-array" tool [8] only provides the 60-mer sequences and EST accession numbers from which the reporters were designed, without detailed or up-to-date annotations. There was therefore a need to develop a strategy for annotation, and thereby build a database of annotations for this maize microarray, as well as similar custom arrays.

The maize B73 genome sequence was released in November 2009 [9], and this provided the opportunity to locate the reporters on the genome sequence, and provide functional annotations. Each reporter is intended to report the expression of a single gene unambiguously. However, since the reporters were designed from ESTs from different maize lines before a reference genome sequence was available [7], redundancy on the array, as well as imperfect reporter matches were expected.

Version 1 and Version 2 Agilent 22 K arrays [10,11] were precursors for the 44 K Agilent-016047 array. Version 1 was designed from the December 2003 maize EST assembly of MaizeGDB and was made up of 21,782 reporters. More than 80% of these reporters were also included in Version 2 plus ~3,000 new reporters, designed from maize sequences in GenBank. Of the 20,963 gene features on Version 2, ~13,000 were sense strand reporters and ~5,000 antisense strand reporters.

For the Agilent-016047 44 K array, an updated set of 60-mer reporters were designed using Picky 2.0 [12]. The reporter set mainly consists of validated reporters from the two precursor maize arrays described above and validated reporters from anther expressed genes detected using a spotted 70-mer array format (containing reporters to about 35,000 maize genes) [10,13]. Additional gene reporters were based on release 16.0 of the TIGR Maize Gene Index as well as cDNA or EST sequences from GenBank (that were at the time not yet in the TIGR Maize Gene Index assembly) [7]. According to Ma *et al. *[7], the 42,034 maize gene reporters represent ~39,000 sense transcripts including a subset of genes with multiple reporters, and ~500 antisense transcripts. In addition to the 42,034 maize gene reporters, the array also contains internal quantitative 'spike-in' controls of non-maize sequences, which were not annotated in this study.

The aims of this study were to annotate the reporter set of the Agilent-016047 microarray by: (i) locating each reporter on the maize B73 genome sequence; (ii) associating each reporter to the transcript of a single gene, if possible; and (iii) assigning functional annotations to the gene represented by each reporter. Our results revealed that we could not associate all of the reporters with a single transcript with high confidence, and therefore we built a database http://MaizeArrayAnnot.bi.up.ac.za, which provides confidence scores of the genomic positions and functional annotations of reporters on the Agilent-016047 Maize array. Our annotation strategy provides guidelines for annotation of custom-designed microarray slides where partial EST information is available, and this resource will therefore be useful to maize researchers, and other researchers using custom arrays.

## Construction and content

### Data sources

The gene list for the Agilent Maize Gene Expression Microarray 4 × 44 K (design ID 016047) was downloaded from Agilent's eArray tool [8] containing a reporter ID, a 60-mer reporter sequence and an EST accession number for each of the 42,034 reporters on the microarray. EST sequence information for 34% of the reporters was available on GenBank, and BioPython [14] was used to extract sequence and other relevant information from individual GenBank files. For an additional 31% of the reporters, EST sequences were obtained from the Walbot laboratory. For the remaining 35% of the reporters, no EST sequences were available, since these are likely to be derived from proprietary sources. The cDNA sequences (in FASTA format), their transcript start and end positions on the B73 RefGen v2 genome sequence as well as InterPro and Gene Ontology (GO) annotations for genes, were downloaded from the maizesequence.org FTP site [15]. Only the protein coding transcripts in the B73 RefGen v2 Working Gene Set (WGS) were used (88,611 cDNAs representing 63,331 genes). We chose to use the WGS and not the Filtered Gene Set (FGS) since it was more inclusive of transcripts that could have been used in the reporter design. The FGS (63,540 transcripts; 39,656 genes) is a subset of the WGS in which transcripts that are "probable pseudogene", "possible transposon", "contamination" or "low confidence" have been filtered out.

The maize B73 RefGen v2 genome sequence (sequences of all 10 chromosomes, in FASTA format) were downloaded from the maizesequence.org FTP site [15]. Lastly, the maize core bin markers [16] and corresponding B73 RefGen v2 base pair positions were retrieved from MaizeGDB [17]. All sets of data were downloaded in December 2010/January 2011.

### Genomic Annotation

**Figure 1:**
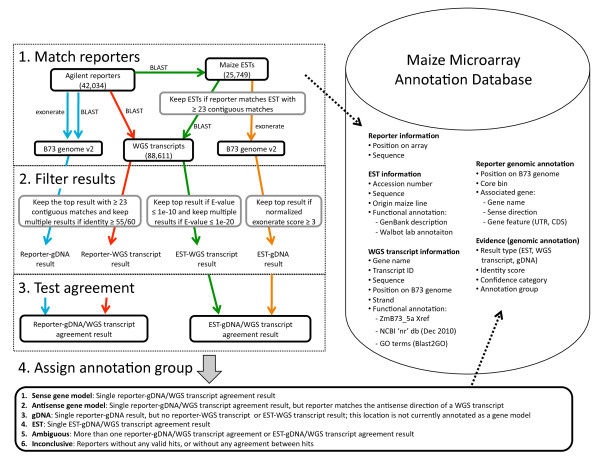
**Strategy followed to assign genomic and functional annotations to the reporters on the Agilent-016047 maize microarray**. Using BLASTN and exonerate software, the 42,034 60-mer reporters were matched to available EST sequences, the maize B73 RefGen v2 genome and the WGS predicted transcripts. BLASTN and exonerate results were filtered and compared to test agreement between EST, WGS transcript and gDNA hits. Based on the agreement analysis, one of six genomic annotation groups was assigned for each reporter. Functional annotations of reporters were based on the functional annotations of their corresponding WGS transcripts. The data has been made accessible from the Maize Microarray Annotation Database http://MaizeArrayAnnot.bi.up.ac.za/.

Figure 1 outlines the strategy that was followed to obtain genomic annotations for each reporter on the Agilent-016047 microarray. All nucleotide sequences were searched against target datasets using the BLASTN algorithm version 2.2.18 [18]. For BLASTN searches of the 60-mer reporter sequences against ESTs, the WGS transcripts and gDNA (B73 RefGen v2), the word size parameter was set to 23 and gaps were not allowed. This cut-off was chosen based on a study that showed that matches of ≥ 23 contiguous nucleotides yielded hybridization signals under stringent conditions in more than 90% of a set of Agilent reporters [19]. Thus, the identity out of 60, rather than the E-value, was used as the measure of similarity for BLASTN searches with the reporters. We also carried out BLASTN searches with EST sequences, and in these cases E-values were used. All BLAST results were stored in a relational database. The parameters used for the BLAST searches are shown in Additional file 1.

Three sets of BLASTN hits were stored for each reporter (Figure 1). Firstly, BLASTN of the reporter was implemented against the genome sequence (B73 RefGen v2), and the top BLASTN hit (if ≥ 23 contiguous matches), or multiple BLASTN hits (if ≥ 23 contiguous matches and identity ≥ 55/60) was called the 'reporter-gDNA result'. The reporters were also searched against the genome sequence using "exonerate" [20] to detect reporters that spanned introns. The parameters for exonerate are shown in Additional file 2. In cases where reporters had positive exonerate matches (and ≥ 23 contiguous matches) to the genome sequence, this result was recorded as the 'reporter-gDNA result'. Secondly, BLASTN of the reporter was implemented against the WGS transcripts, and the top BLASTN hit (if ≥ 23 contiguous matches), or multiple BLASTN hits (if ≥ 23 contiguous matches and identity ≥ 55/60) was called a 'reporter-WGS transcript result'. Thirdly, after confirming that the reporter matched its corresponding EST listed in the Agilent eArray database (≥ 23 contiguous matches), the top BLASTN hit (if E-value ≤ 1e-10), or multiple BLASTN hits (if E-value ≤ 1e-20) of the EST against the WGS transcript dataset was called an 'EST-WGS transcript result' (BLASTN parameters in Additional file 1). These BLASTN cut-offs were selected based on a previous study where the same cut-offs were used to align ESTs to predicted maize cDNAs [21]. The ESTs were also searched against the gDNA using exonerate (parameters in Additional file 2), and matches with a normalised score of at least 3 (calculated by dividing the exonerate raw score by the query EST sequence length) [22] were recorded as the 'EST-gDNA result'.

The next step was to determine if there was agreement between the reporter-gDNA result and the reporter-WGS transcript result, in other words whether the WGS transcript that the reporter matched was derived from the same gDNA position that the reporter matched. This was recorded as the reporter-gDNA/WGS agreement result (Figure 1). Similarly, we tested whether the EST-gDNA result and EST-WGS transcript result were in agreement, and recorded this as the EST-gDNA/WGS agreement result (Figure 1).

Finally, the reporters were placed into one of six annotation groups, informed by sequence matching and agreement results described above and whether the genomic position of the reporter overlapped with one or more gene models in the sense or antisense direction (Figure 1). The annotation groups were:

(i) Annotated by sense gene model: Reporters that match a WGS transcript and genomic location of the same gene model (single reporter-gDNA/WGS agreement result);

(ii) Annotation by antisense gene model: Reporters that match a transcript and genomic location of the same gene model, but align to the antisense direction of the transcript (single reporter-gDNA/WGS agreement result);

(iii) Annotation by gDNA: Reporters that match a unique location on the maize B73 genome, but this location is not currently annotated as a gene model (single genomic result);

(iv) Annotation by EST: Reporters that do not match a WGS transcript, but that are derived from an EST that matches a WGS transcript and its genomic location (single EST-gDNA/WGS agreement result);

(v) Ambiguous annotation: Reporters with more than one sense gene model, antisense gene model or EST result (More than one reporter-gDNA/WGS transcript agreement or EST-gDNA/WGS transcript agreement result);

(vi) Inconclusive annotation: Reporters that match more than one transcript, but not the genomic location of the corresponding gene models. Reporters that match more than one genomic location, but no corresponding transcripts. Reporters with no valid hits.

### Functional annotation

The "reporter-WGS transcript result" for each reporter (described above) was used to assign a functional annotation to each reporter. The functional annotations for each of the 88,611 cDNA sequences in the WGS of the B73 RefGen v2 genome sequence were obtained by BLASTX [18] searches (with default parameter settings; Additional file 1) against the NCBI non-redundant peptide database (nr). The top three hits (and corresponding statistics) were stored in a relational database. Blast2GO [23] was used to associate each WGS transcript (and therefore the corresponding reporters) with Gene Ontology (GO) terms, using default settings.

### Database and web interface

The Maize Microarray Annotation Database interface was written using Turbogears [24], a Python web application framework. A central MySQL database is used to store sequence and annotation information. SQLAlchemy [25], an object relational mapper for Python and toolkit for SQL, is implemented within the Maize Microarray Annotation Database when a user queries the database.

### Integration with the MaizeGDB genome browser

An annotation file with the genomic positions for each reporter that could be matched to the genome was generated (Additional file 3). This can be uploaded to the MaizeGDB genome browser [26] and viewed as an annotation track in the context of the B73 RefGen v2 genome sequence.

### Reporters with expression in maize leaf material

Reporters "with measurable signal" were identified as those with a signal to noise ratio (SNR) > 3 in at least one of fifty Agilent-016047 microarrays hybridized with cDNA from maize leaves of a segregating population (data not shown).

## Utility and Discussion

### Genomic annotation groups

Table 1 shows the breakdown of the annotation groups of the 42,034 reporters on the maize Agilent-016047 microarray, as determined by our strategy outlined in Figure 1. Importantly, 27,998 reporters (67%) were annotated by gene model in the sense or antisense direction, which means that they correspond to a transcript with a defined gDNA position. Approximately half of the reporters in this group mapped to UTR regions, and the rest to coding regions. A number of these reporters (1,554) were shown using exonerate software [20] to span introns.

**Table 1:** Number of reporters placed in the genomic annotation groups of the maize Agilent-016047 microarray

Annotation groups	**Number of reporters**^**a**^	**Reporters with Signal/Noise > 3 (maize leaves)**^**b**^	**Proportion of reporters with Signal/Noise > 3**^**c**^
sense gene model	23668 (56.3%)	20752	88%
antisense gene model	4330 (10.3%)	2470	57%
gDNA	1549 (3.7%)	985	64%
EST	3390 (8.1%)	1920	60%
ambiguous	2608 (6.2%)	2208	84%
inconclusive	6489 (15.4%)	4038	61%
Total	42034 (100%)	32373	77%

**^a ^**The number and percentage of reporters in each annotation group.

**^b ^**Hybridization to fifty Agilent-016047 arrays by maize leaf cDNA from a segregating population

**^c ^**Percentage calculated from the number of reporters with signal/noise > 3 in maize leaves divided by the total number of reporters in each annotation group.

The reporters annotated by gene model in the sense or antisense direction represent 46.7% of the genes in the B73 maize Filtered Gene Set (FGS). Within this group, there were 4,330 reporters that aligned to the antisense direction of a gene model (Table 1). Natural antisense transcripts (NATs) contain sequences complementary to the sense transcripts of protein-coding genes [27]. Between 7 and 30% of genes in animal and plant genomes encode overlapping cis-NATs [27]. Many NATs are conserved, implying regulatory functions for these transcripts in gene expression. According to Ma *et al. *[10], 14.3% of the pollen transcriptome consists of detectable antisense transcripts. It should be borne in mind that, in some cases, a reporter with an antisense annotation could in fact correspond to a sense transcript if the EST from which it was designed was incorrectly oriented or the genomic annotation was in the wrong strand. These errors are expected to be corrected in future annotation versions of the maize B73 genome sequence.

The 1,549 reporters "annotated by gDNA" (Table 1) represent reporters that had significant matches to the maize genome sequence but did not match current transcripts in the WGS of the maize B73 RefGen v2. These reporters will possibly be linked to gene models in future versions of the B73 genome due to improvements in gene prediction algorithms or availability of mRNA-seq data from different tissues of maize B73 plants. Although these reporters are not currently associated with functional annotations, their placement on the B73 genome sequence is useful for eQTL studies in maize. This category of reporters showed a lower proportion (64%) with measurable expression in maize leaves compared to reporters annotated by sense gene model (88%; Table 1).

The 3,390 reporters "annotated by EST" (Table 1) were derived from ESTs that showed sequence similarity to a WGS transcript from B73 (E-value < 1e^-10^), however the reporter itself did not have a significant hit to the WGS transcript. The reporters on the maize Agilent-016047 microarray were designed from ESTs from various maize lines (Additional file 4). Reporters "annotated by EST" are most likely derived from maize lines other than B73, although the source is not known for all reporters since this information could be retrieved for only 34% of the reporters (Additional file 4A). The region of the transcript that corresponds to the reporter is therefore predicted to be divergent between B73 and the line from which the reporter was derived.

The 2,608 "ambiguous" reporters (Table 1) each represent more than one gene model, which are mostly members of the same gene family. Interpretation of expression data from these reporters should be done with caution, as it is possible that the signal is due to cross hybridization from more than one family member. As an example, reporter A_92_P037799 represents four members of the cytochrome P450 gene family on chromosomes 2, 3, 6 and 8, as shown in the multiple sequence alignment (Additional file 5).

There were 6,489 reporters with "inconclusive annotation" (Table 1), and thus interpretation of expression data from these reporters should be made with caution. This group contained a relatively low proportion of reporters with signal in a maize leaf microarray experiment conducted in our laboratory (see Methods), namely 61% compared to 88% of reporters "annotated by sense gene model" (Table 1). Re-sequencing of six maize lines from China identified several hundred genes that were not present in B73, but could be annotated as plant proteins [28], and therefore it is possible that reporters with "inconclusive annotation" may represent transcribed genes from other maize lines. A subset of the reporters with inconclusive annotation and no hits against the B73 genome sequence had EST sequences available (1,727) and these were searched against GenBank using BLASTX. Only 892 had significant hits (E-value ≤ 1e-10) and 553 matched plant proteins.

Prior to our work, the reporters on the Agilent-016047 maize array could be visualized in the context of the B73 maize genome sequence at MaizeGDB [29] based on the Walbot laboratory annotations. However, there are several limitations of this annotation track, namely: (i) the positions given are based on RefGen v1, whereas the sequence is RefGen v2; (ii) the positions are based on MegaBLAST hits to the gDNA, but no matches to transcripts are given; (iii) the reporters are named using a UID which is different from the Agilent e-array ID; and (iv) three confidence categories are given, however some reporters have up to 500 hits. Therefore we have produced an updated annotation track that is compatible with MaizeGDB (Additional file 3) that reports the positions of all reporters on the array except those with inconclusive annotation or annotation by EST. An example of three reporters that match one gene model is shown in Additional file 6.

### Maize Microarray Annotation Database

The Maize Microarray Annotation Database has an interactive web interface http://MaizeArrayAnnot.bi.up.ac.za/, providing the user with three main functionalities namely "Search Agilent slide", "BLAST sequences" and "Get sequences from GenBank" (Additional file 7). Most users are likely to use the "search Agilent slide" function, since they would be interested in downloading annotations for a list of reporters that are differentially expressed in a microarray experiment. In order to search the Agilent slide, the user can provide Reporter IDs, EST Accession numbers or gene names. The outputs from a query are reporter information, EST information, gene information, genomic and functional annotation information as well as the evidence for the annotation results. The following can be downloaded: DNA sequences (reporter, EST or WGS transcript sequences in FASTA format), a table with all annotation information, and/or multiple sequence alignments. Searching by WGS gene name makes it possible to see whether there is more than one reporter for a gene. On average, there are ~1.6 reporters per gene. Users can also retrieve nucleotide sequences by submitting GenBank accession numbers for ESTs, or BLAST sequence(s) against the Agilent slide to identify which reporters represent the query sequence best.

### Case studies

Table 2 gives a selection of five publications in which the Agilent-016047 array has been used, with an indication of how many reporters gave a measurable signal according to the authors. Lack of a signal may be due to tissue specific expression, genotype differences, or poor reporter design. Ma *et al. *[7] used this microarray to study the expression profiles of maize anther and pollen ontogeny. They found that more than 24,000 different transcript types were expressed, and that each anther stage expressed ~10,000 constitutive and ~10,000 or more transcripts restricted to one or a few stages in anther development. Casati *et al. *[30] measured transcriptome changes between RNAi transgenic maize lines and a UV-B tolerant B73 control line, using this Agilent slide. Approximately 26,000 reporters showed expression in adult maize leaves. Skibbe *et al. *[31] hypothesized that Mutator transposon activity reprograms the transcriptomes of developing maize anthers. About 35,000 reporters had signals > 2.6 times the standard deviation of the background (i.e. 99.5% confidence interval), and they concluded that Mu transposition activated by transcriptionally active *MuDR *results in a 25% change in the transcriptome. Wang *et al. *[4] hypothesized that the male sterile 8 mutation (ms8) of maize disrupts the temporal progression of the transcriptome. They found that fertile anthers exhibit an unexpectedly high transcript complexity; there were 27,400 constitutively expressed transcripts, 2,143 stage-specific transcripts and 2,484 transcripts that were expressed at two stages, giving ~32,000 transcripts in total that were expressed over a 90-h period. Lastly, Rajhi *et al. *[32] used this array and laser microdissection to identify transcripts expressed in maize root cortical cells during lysigenous aerenchyma formation.

**Table 2:** List of studies using the Agilent-016047 maize microarray

Publication	Maize Tissue	Signal	No Signal	Criteria to define a 'Signal'
Ma *et al. *[7]	anther and pollen	> 24 K	< 18 K	3 out of 4 hybridization signals > background (99% confidence)
Casati *et al. *[30]	adult leaves	26 K	16 K	median reporter intensity > background
Skibbe *et al. *[31]	developing anthers	30 K	14 K	reporter intensity > 2.6 × SD of background
Wang *et al. *[4]	fertile anthers	32 K	10 K	not specified
Rajhi *et al. *[32]	roots	no info	no info	not specified
Current study	adult leaves	32 K	10 K	signal/noise > 3

We analysed expression data from hybridization of maize leaf cDNA from a segregating population to fifty Agilent-016047 arrays to assess the number of reporters with measurable signal in our hands. The data showed that ~32,000 reporters had a consistent signal to noise ratio (SNR) greater than 3, whereas ~10,000 reporters were deemed non-hybridizing to leaf transcripts (Table 2). These six studies demonstrate that in all cases a large proportion of the reporters on the Agilent-016047 arrays give measurable signals in tissues as diverse as anthers, leaves and roots.

The questions are, however, how many genes are represented by these reporters and how much confidence is there in their annotations? To address these questions, we extracted tables of differentially expressed reporters reported in these studies and annotated the reporters using our Maize Microarray Annotation Database (Table 3). Most of the data tables have the majority of reporters annotated with high confidence by a single sense or antisense gene model (59-86% of reporters in each data table, Table 3). However, 3-9% of reporters have ambiguous annotations, and thus their hybridization signals could be due to cross-hybridization between gene family members (Table 3). This is of particular relevance in the data table S4 from Ma *et al. *[7] which was a selection of reporters corresponding to Zinc finger-related proteins, where 9% of reporters were "ambiguous". Each data table contained reporters with inconclusive annotations. The data table which appears to be the exception is the study of gene expression in anthers of the *ms8 *mutant in which only 38% of reporters were annotated by sense gene model, and this table had a higher proportion of antisense, EST and inconclusive annotation reporters (14%, 13% and 25%, respectively). This may reflect a difference in the biology of this experiment compared to the other experiments.

**Table 3:** Case studies using the Maize Microarray Annotation Database

Reference	Description *	Table (see Reference)	Total	Genomic Annotation Groups^#^					
				
				GMS	GMA	G	E	AM	I
Ma *et al. *[4]	a	Table S3	2285	1614 (70.6%)	141 (6.2%)	54 (2.4%)	108 (4.7%)	148 (6.5%)	220 (9.6%)
Ma *et al. *[4]	b	Table S4	281	209 (74.4%)	11 (3.9%)	7 (2.5%)	13 (4.6%)	27 (9.6%)	14 (5.0%)
Casati *et al. *[30]	c	Table S1	2092	1373 (65.6%)	142 (6.8%)	58 (2.8%)	111 (5.3%)	134 (6.4%)	274 (13.1%)
Skibbe *et al. *[31]	d	Table S1	449	329 (73.3%)	6 (1.3%)	18 (4.0%)	16 (3.6%)	35 (7.8%)	45 (10.0%)
Skibbe *et al. *[31]	e	Table S1	399	279 (69.9%)	15 (3.8%)	16 (4.0%)	18 (4.5%)	23 (5.8%)	48 (12.0%)
Wang *et al. *[4]	f	Table S3	416	159 (38.2%)	58 (13.9%)	22 (5.3%)	57 (13.7%)	14 (3.4%)	106 (25.5%)
Rajhi *et al. *[32]	g	Table S2	239	177 (74.1%)	15 (6.3%)	5 (2.1%)	10 (4.2%)	12 (5.0%)	20 (8.4%)
Rajhi *et al. *[32]	h	Table S3	336	278 (82.7%)	7 (2.1%)	8 (2.4%)	14 (4.2%)	15 (4.5%)	14 (4.2%)

* Description

a = Transcripts differentially expressed between meiotic and post-meiotic stages

b = Zinc finger-related proteins

c = Transcripts that are expressed differentially between mbd101 and chc101 RNAi transgenic plants and WT non transgenic siblings under control and/or UV-B conditions.

d = Up-regulated genes between Mu-active vs inactive lines (Mitotic stage)

e = Down-regulated genes between Mu-active vs inactive lines (Mitotic stage)

f = 1.0 mm stage-specific genes of *ms8 *anthers expressed at later stages in normal anthers

g = Genes up-regulated in maize root cortex during aerenchyma formation

h = Genes down-regulated in maize root cortex during aerenchyma formation

**^# ^**Genomic annotations:

GMS = Annotation by sense gene model

GMA = Annotation by antisense gene model

G = Annotation by gDNA (Genomic position)

E = Annotation by EST

AM = Ambiguous annotation

I = Inconclusive annotation

We suggest that annotation of reporters with the Maize Microarray Annotation Database can be useful for refining lists of "differentially expressed" reporters for subsequent global analyses (e.g. GO enrichment using tools such as MADIBA [33]). In addition, the database is also essential to confirm the annotation of candidate genes identified from a microarray experiment before detailed functional analyses (e.g. gene knockouts) are carried out. To this end, we have provided, as Additional files 8, 9, 10, 11, 12, 13, 14 and 15, our annotations of the data tables from the case studies listed in Table 3.

The importance of correct annotation of microarrays is illustrated by the study of Gertz *et al. *[34] who performed a similar analysis on the 44 K Agilent human expression arrays and found that many reporters had inconclusive annotations. Out of 42,683 reporters, 25,505 (60%) were considered 'fully valid' according to their analyses. In another study, an Agilent mouse 44 K array was re-annotated resulting in improved annotations for more than 10,000 reporters on the array [35]. Furthermore, gene models are constantly being updated as new experimental and annotation data accumulates. Therefore re-annotation of reporters is required as illustrated by a study in which a dozen mammalian GeneChip arrays were re-annotated [36]. This would be of particular importance in maize where the genome sequence was recently released [9] and is currently only at version 2 of annotation.

## Conclusions

A reporter-by-reporter validation of the 4 × 44 K Agilent-016047 maize microarray was performed. In total, 71% of the reporters correspond to a transcript with a defined gDNA position and represent 46.7% of the genes in the B73 FGS. All results have been included in a database http://MaizeArrayAnnot.bi.up.ac.za/, which provides confidence scores of the genomic positions and functional annotations of reporters on the Agilent-016047 Maize array. The database facilitates interpretation of maize gene expression data. Scientists embarking on expression profiling in maize are likely to find this array an attractive option, since the combination of our annotation database with established analysis methods [37] facilitates data interpretation. In addition, our strategy can be applied when annotating any custom-designed array from a species for which the genome sequence is available.

## Availability and requirements

The Maize Microarray Annotation Database is publicly available at http://MaizeArrayAnnot.bi.up.ac.za/.

## List of abbreviations

BLAST: Basic Local Alignment Search Tool; eQTL: Expression Quantitative Trait Locus; EST: Expressed Sequence Tag; FGS: Filtered Gene Set; gDNA: Genomic Deoxyribonucleic Acid; GO: Gene Ontology; NAT: Natural Antisense Transcript; WGS: Working Gene Set.

## Competing interests

The authors declare that they have no competing interests.

## Authors' contributions

NC built the database, analysed the data, and drafted the manuscript. DKB initiated the study, contributed to the strategy, database design and analysis, and helped to draft the manuscript. ZM contributed to the strategy and database design and helped to edit the manuscript. All authors have read and approved the final manuscript.

## Supplementary Material

Additional file 1 **BLAST parameters used for annotation of the Agilent-016047 maize microarray**. Click here for file

Additional file 2 **Parameters used for exonerate analysis of the Agilent-016047 maize microarray reporters and ESTs against the B73 maize genome sequence**. Click here for file

Additional file 3 **Genomic positions for each reporter that could be matched to the genome**. Annotation file with the genomic positions for each reporter that can be uploaded to the MaizeGDB genome browser. Click here for file

Additional file 4 **Sources of maize ESTs**. (A) ESTs (39,174) from which reporters on the Agilent-016047 microarray were designed. (B) Sources of ESTs with GenBank annotations (13,640). Click here for file

Additional file 5 **Example of a reporter in the "ambiguous" annotation group**. Multiple alignment of reporter A_92_P037799 with corresponding parts of four maize cytochrome P450 cDNAs. Click here for file

Additional file 6 **Screenshot of the B73 RefGen v2 genome browser at MaizeGDB**. Three Agilent reporters (A_92_P007469, A_92_P025231, A_92_P040586) are linked to gene model GRMZM2G089944 on chromosome 3. Click here for file

Additional file 7 **Screenshot of the Maize Microarray Annotation Database**. The Maize Microarray Annotation Database enables users to retrieve reporter-specific and global information regarding the reporters on the Agilent-016047 microarray. Click here for file

Additional file 8 **Re-annotation of transcripts differentially expressed between meiotic and post-meiotic stages**. Ma *et al. *[7]; Table S3. Click here for file

Additional file 9 **Re-annotation of expressed zinc finger-related proteins**. Ma *et al. *[7]; Table S4. Click here for file

Additional file 10 **Re-annotation of transcripts that are expressed differentially between mbd101 and chc101 RNAi transgenic plants and WT non-transgenic siblings under control and/or UV-B conditions**. Casati *et al. *[30]; Table S1. Click here for file

Additional file 11 **Up-regulated genes between Mu-active versus inactive lines (Mitotic stage)**. Skibbe *et al. *[31]; Table S1. Click here for file

Additional file 12 **Down-regulated genes between Mu-active versus inactive lines (Mitotic stage)**. Skibbe *et al. *[31]; Table S1. Click here for file

Additional file 13 **Re-annotation of 1.0 mm stage-specific genes of *ms8 *anthers expressed at later stages in normal anthers**. Wang *et al. *[4]; Table S3. Click here for file

Additional file 14 **Re-annotation of genes up-regulated in maize root cortex during aerenchyma formation**. Rajhi *et al. *[32]; Table S2. Click here for file

Additional file 15 **Re-annotation of genes down-regulated in maize root cortex during aerenchyma formation**. Rajhi *et al. *[32]; Table S3. Click here for file
