# Supplementary material for: Maize microarray annotation database
Source: Plant Methods. 2011 Oct 1;7:31. doi: 10.1186/1746-4811-7-31 (PMC3198759; doi:10.1186/1746-4811-7-31)
Supplement: Additional file 1 — BLAST parameters used for annotation of the Agilent-016047 maize microarray. [file 1746-4811-7-31-S1.PDF]

Additional File 1. BLAST parameters used for annotation of the Agilent-016047 maize microarray

BLAST parameter	reporters vs (i) ESTs; (ii) WGS transcripts; (iii) gDNA	ESTs vs WGS transcripts	WGS transcripts vs GenBank (functional annotation)
**Program name**	BLASTN	BLASTN	BLASTX
**Word size**	23	11 (default)	3 (default)
**Filter query sequence for low-complexity subsequences**	TRUE (default)	TRUE (default)	TRUE (default)
**Gapped alignment**	FALSE	TRUE (default)	TRUE (default)
**Initial penalty for opening a gap**	NA (no gaps allowed)	5 (default)	11 (default)
**Penalty for each gap character**	NA (no gaps allowed)	2 (default)	1 (default)
**Threshold expectation value for keeping alignments**	10 (default); own filtering on identity out of 60	10^{-10}	10^{-10}
**Protein similarity matrix**	not used in BLASTN	not used in BLASTN	BLOSUM62 (default)
