# Supplementary material for: Maize microarray annotation database
Source: Plant Methods. 2011 Oct 1;7:31. doi: 10.1186/1746-4811-7-31 (PMC3198759; doi:10.1186/1746-4811-7-31)
Supplement: Additional file 2 — Parameters used for exonerate analysis of the Agilent-016047 maize microarray reporters and ESTs against the B73 maize genome sequence. [file 1746-4811-7-31-S2.PDF]

Additional file 2. Parameters used for exonerate analysis of the Agilent-016047 maize microarray reporters and ESTs against the B73 maize genome sequence

Exonerate parameter	Value	Description
**alignment model**	est2genome	This model is similar to the affine:local model, but it also includes intron modelling on the target sequence to allow alignment of spliced to unspliced coding sequences for both forward and reversed genes
**bestn**	1	Report the best N results for each query (only results scoring better than the score threshold will be reported)
**minintron**	30 (default)	Minimum intron length limit
**maxintron**	100000	Maximum intron length limit
**raw score**	100 (default minimum score)	The sum of transition scores used in the dynamic programming. The maximum score depends on the query sequence length.
**gapopen**	-12 (default)	This is the gap open penalty
**gapextend**	-4 (default)	This is the gap extension penalty
**mismatch**	-9 (default)	Mismatch penalty
