# Supplementary figures and images for: Maize microarray annotation database
Source: Plant Methods. 2011 Oct 1;7:31. doi: 10.1186/1746-4811-7-31 (PMC3198759; doi:10.1186/1746-4811-7-31)

B.

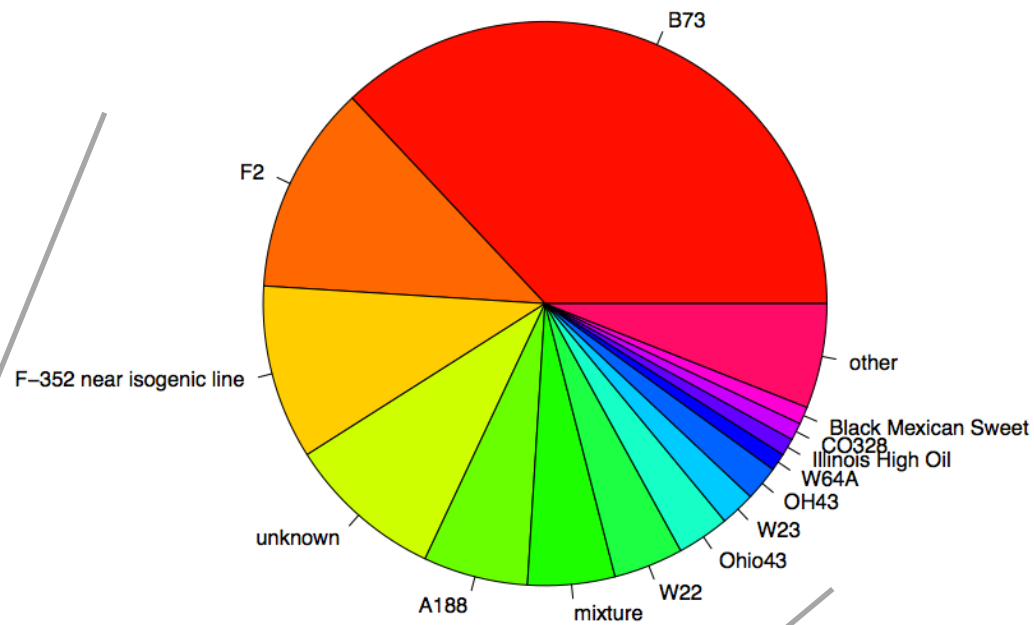

A.

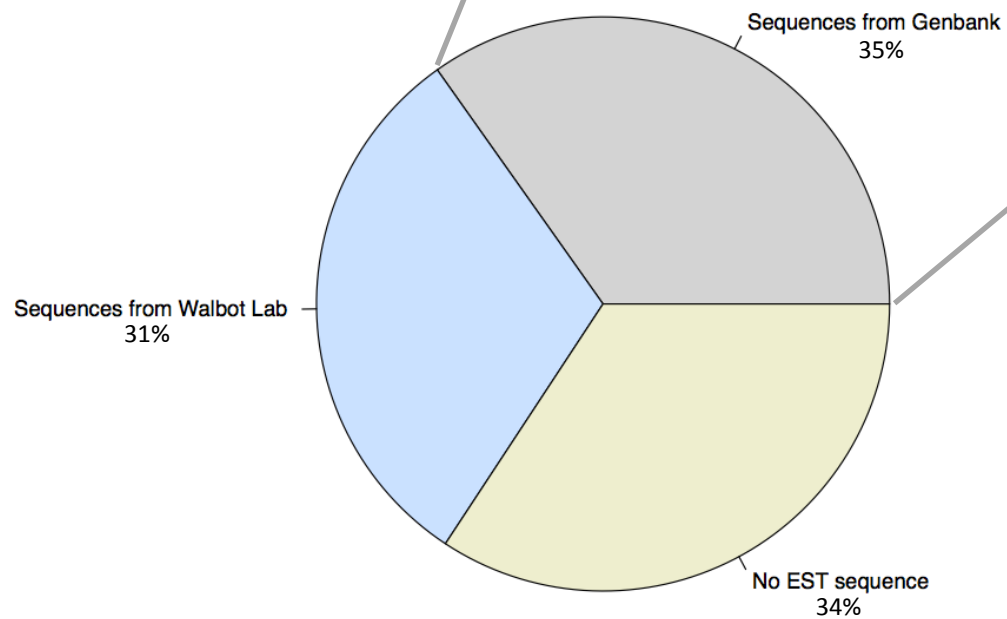

Supplement: Additional file 4 — Sources of maize ESTs. (A) ESTs (39,174) from which reporters on the Agilent-016047 microarray were designed. (B) Sources of ESTs with GenBank annotations (13,640). [file 1746-4811-7-31-S4.PDF]
