# Supplementary material for: Maize microarray annotation database
Source: Plant Methods. 2011 Oct 1;7:31. doi: 10.1186/1746-4811-7-31 (PMC3198759; doi:10.1186/1746-4811-7-31)
Supplement: Additional file 6 — Screenshot of the B73 RefGen v2 genome browser at MaizeGDB. Three Agilent reporters (A_92_P007469, A_92_P025231, A_92_P040586) are linked to gene model GRMZM2G089944 on chromosome 3. [file 1746-4811-7-31-S6.PDF]

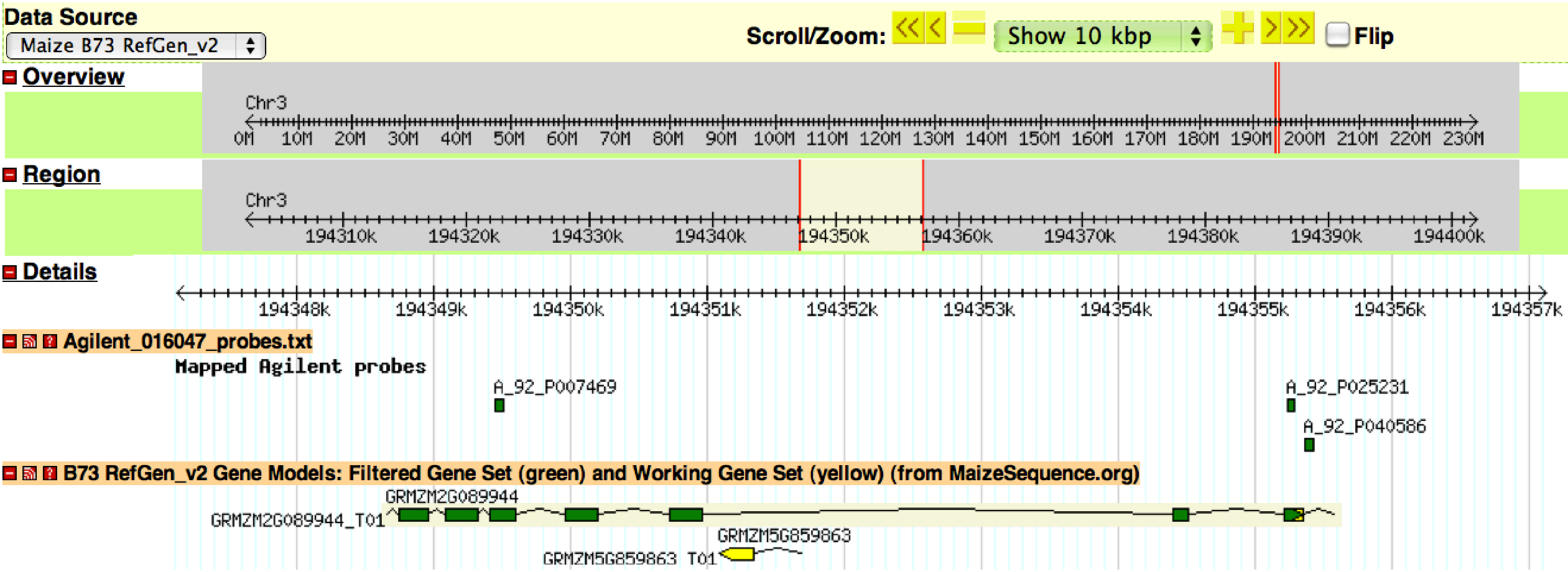
Data Source
Maize B73 RefGen_v2
Scroll/Zoom:
Show 10 kbp
Flip
Overview
Chr3
0M 10M 20M 30M 40M 50M 60M 70M 80M 90M 100M 110M 120M 130M 140M 150M 160M 170M 180M 190M 200M 210M 220M 230M
Region
Chr3
194310k 194320k 194330k 194340k 194350k 194360k 194370k 194380k 194390k 194400k
Details
194348k 194349k 194350k 194351k 194352k 194353k 194354k 194355k 194356k 194357k
Agilent_016047_probes.txt
Mapped Agilent probes
A_92_P007469
A_92_P025231
A_92_P040586
B73 RefGen_v2 Gene Models: Filtered Gene Set (green) and Working Gene Set (yellow) (from MaizeSequence.org)
GRMZM2G089944
GRMZM2G089944_T01
GRMZM5G859863
GRMZM5G859863_T01
