# Supplementary material for: Maize microarray annotation database
Source: Plant Methods. 2011 Oct 1;7:31. doi: 10.1186/1746-4811-7-31 (PMC3198759; doi:10.1186/1746-4811-7-31)
Supplement: Additional file 7 — Screenshot of the Maize Microarray Annotation Database. The Maize Microarray Annotation Database enables users to retrieve reporter-specific and global information regarding the reporters on the Agilent-016047 microarray. [file 1746-4811-7-31-S7.PDF]

Maize Microarray Annotation Database

Home Page | Search Agilent Slide | Blast sequences | Get sequences from GenBank

Select/ Unselect All ☑	Agilent ID	EST Accession Number	Annotation Group+	Result Number	Position Type#	Identity Score#	ZmB73 Chr	Start (bp)	Stop (bp)	Core Bin	Zm GeneID	Sense Direction	Gene Feature	Zm Gene Descriptor	Blast2GO Description
☑	A_92_P037670	TC308201	antisense gene model	1/1	cDNA	60.0	chr 5	180868777	180878474	5.05	GRMZM2G399952	no	CDS span intron (size=98)	hypothetical protein LOC100274541	-
☑	A_92_P037674	TC295391 (no seq)	sense gene model	1/1	cDNA	60.0	chr 3	174688276	174694367	3.06	GRMZM2G002626	yes	CDS	hypothetical protein LOC100383265	-
☑	A_92_P024994	TC285889 (no seq)	gDNA	1/1	gDNA	60.0	chr 1	180624583	180624524	1.06	-	-	-	-	-
☑	A_92_P017983	TC300604 (no seq)	ambiguous	1/3	cDNA	60.0	chr 10	101157262	101160185	10.04	GRMZM5G881323	no	UTR	Putative uncharacterized protein	ethylene receptor
	A_92_P017983	TC300604 (no seq)	ambiguous	2/3	cDNA	60.0	chr 10	101157176	101161092	10.04	GRMZM2G420801	yes	CDS	ethylene receptor homolog2	ethylene receptor
	A_92_P017983	TC300604 (no seq)	ambiguous	3/3	cDNA	58.0	chr 2	103960946	103965539	2.05	GRMZM2G089010	yes	CDS	hypothetical protein LOC100193682	ethylene receptor
☑	A_92_P037687	TC301758	EST	1/1	EST	e-val= 0.0	chr 1	35239930	35250929	1.03	GRMZM2G030422	no info	CDS overlaps intron	hypothetical protein LOC100276114	-
☑	A_92_P037705	DN559245	EST	1/1	EST	e-val= 2e-70	chr 8	131002615	131006857	8.05	GRMZM2G477741	no info	NA	metal tolerance protein A2	metal tolerance protein a2
☑	A_92_P026516	TC310712 (no seq)	sense gene model	1/1	cDNA	60.0	chr 8	138861203	138862890	8.05	GRMZM2G013448	yes	UTR	1-aminocyclopropane-1-carboxylate oxidase 1	1-aminocyclopropane-1-carboxylic acid oxidase
☑	A_92_P026517	AW331475	inconclusive	0	no result	-	-	-	-	-	-	-	-	-	-
☑	A_92_P039116	TC300271 (no seq)	sense gene model	1/1	cDNA	60.0	chr 6	162556092	162559012	6.07	GRMZM2G059825	yes	UTR	hypothetical protein LOC100191546	secondary cell wall-related glycosyltransferase family 47
