# Supplementary material for: Maize microarray annotation database
Source: Plant Methods. 2011 Oct 1;7:31. doi: 10.1186/1746-4811-7-31 (PMC3198759; doi:10.1186/1746-4811-7-31)
Supplement: Additional file 5 — Example of a reporter in the "ambiguous" annotation group. Multiple alignment of reporter A_92_P037799 with corresponding parts of four maize cytochrome P450 cDNAs. [file 1746-4811-7-31-S5.PDF]

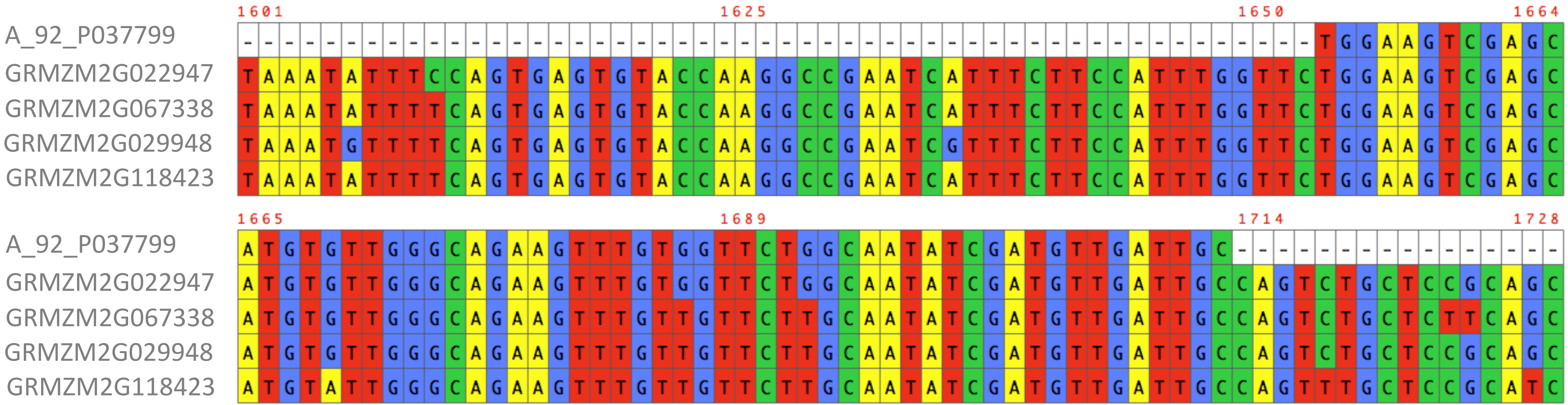

1601 1625 1650 1664
A_92_P037799 --TGGAAGTCGAGC
GRMZM2G022947 TAAATATTTCCAGTGAGTGTACCAAGGCCGAATCATTTCTTCCATTTGGTTCTGGAAGTCGAGC
GRMZM2G067338 TAAATATTTTCAGTGAGTGTACCAAGGCCGAATCATTTCTTCCATTTGGTTCTGGAAGTCGAGC
GRMZM2G029948 TAAATGTTTTCAGTGAGTGTACCAAGGCCGAATCGTTTCTTCCATTTGGTTCTGGAAGTCGAGC
GRMZM2G118423 TAAATATTTTCAGTGAGTGTACCAAGGCCGAATCATTTCTTCCATTTGGTTCTGGAAGTCGAGC
1665 1689 1714 1728
A_92_P037799 ATGTGTTGGGCAGAAGTTTGTGGTTCTGGCAATATCGATGTTGATTGC----------------
GRMZM2G022947 ATGTGTTGGGCAGAAGTTTGTGGTTCTGGCAATATCGATGTTGATTGCCAGTCTGCTCCGCAGC
GRMZM2G067338 ATGTGTTGGGCAGAAGTTTGTTGTTCTTGCAATATCGATGTTGATTGCCAGTCTGCTCTTCAGC
GRMZM2G029948 ATGTGTTGGGCAGAAGTTTGTTGTTCTTGCAATATCGATGTTGATTGCCAGTCTGCTCCGCAGC
GRMZM2G118423 ATGTATTGGGCAGAAGTTTGTTGTTCTTGCAATATCGATGTTGATTGCCAGTTTGCTCCGCATC
